# Xiaoyaosan Decoction Regulates Changes in Neuropeptide Y and Leptin Receptor in the Rat Arcuate Nucleus after Chronic Immobilization Stress

**DOI:** 10.1155/2012/381278

**Published:** 2012-11-13

**Authors:** Shao-Xian Wang, Jia-Xu Chen, Guang-Xin Yue, Ming-Hua Bai, Mei-Jing Kou, Zhong-Ye Jin

**Affiliations:** School of Preclinical Medicine, Beijing University of Chinese Medicine, P.O. Box 83, No. 11, Beisanhuan Donglu, Chaoyang District, Beijing 100029, China

## Abstract

The arcuate nucleus (ARC) in the basal of hypothalamus plays an important role in appetite regulation and energy balance. We sought to investigate the central neuroendocrine mechanism of appetite decrease and weight loss under chronic stress by observing the regulatory effects of Xiaoyaosan decoction in the expression of leptin receptor (*ob-R*) and neuropeptide Y (NPY) in the ARC. Our results showed that bodyweight and food intake of rats in the 21-day stress group increased slower than those of the normal group. Higher contents of Leptin and *ob-R* were noted in the 21-day stress group compared with control rats, while NPY expression was not statistically different. Xiaoyaosan powder can significantly downregulate the contents of leptin and *ob-R* in the hypothalamus of stressed rats. These findings suggest that increase of *ob-R* expression in the ARC is possibly one key central neuroendocrine change for the somatic discomfort. Weight loss and decreased food intake in rats caused by the binding of leptin to *ob-R* in hypothalamus do not appear to utilize the NPY pathway. This study also suggests that *ob-R* in the ARC may act as the target of Xiaoyaosan in regulating the symptoms such as appetite decrease and bodyweight loss under chronic stress.

## 1. Introduction

The body needs timely adjustment of physiological status to adapt to stress. Moderate stress is beneficial to the body; while excessive stress can influence the body's mental and physical health. Studies show that stressful events significantly affect body's feeding behaviors [1] and that, long-term, chronic, and repeated stresses can cause decreased food intake and bodyweight loss in rats [2–6]. Previous experiments of this research team also suggested that chronically stressed rats presented abnormalities of emotions and behaviors such as depression and anxiety, which were mostly accompanied by slow increases of food intake and bodyweight along with other changes [7, 8]. At present, most research focuses on the central neuroendocrine mechanisms of abnormalities of emotions and behaviors such as stress-induced depression and anxiety; while there are few studies on the mechanisms underlying food intake and bodyweight changes under stress.

The hypothalamic nucleus group is required for the regulation of energy balance. Specifically, the ARC in the basilar part of hypothalamus plays an important role in appetite regulation and energy balance. Neuropeptide Y (NPY) is a polypeptide with biological activity composed of 36 amino acids that is widely distributed in the mammalian central and peripheral nervous systems. In hypothalamus, NPY content is the highest; while expression of NPY neurons in hypothalamus is the most in the ARC [9]. Leptin receptor (*ob-R*) belongs to a family of cytokines. The hormone receptor *ob-R* plays a role by binding with specificity of leptin to regulate many physiological functions. Also, *ob-R* is widely distributed in the central nervous system of normal rats and hypothalamus nucleus groups such as the ARC, paraventricular nucleus, ventromedial nucleus, and dorsomedial [10, 11]. Studies show that *ob-R* and NPY coexist in the ARC [12–14] and that the binding of *ob-R* with leptin can influence the synthesis and secretion of NPY and, thus, regulate food intake and energy metabolism [15–17]. Specifically, NPY can enhance appetite and promote food intake. Conversely, binding of *ob-R* can inhibit appetite and decrease food intake. In energy metabolism, feeding behavior and bodyweight, NPY and *ob-R* oppose and assist each other.

Studies showed that NPY can regulate emotional and behavioral changes caused by stress and can induce antistress and antianxiety effects in multiple-stress animal models [18, 19]. The hypothalamic-pituitary-adrenal (HPA) axis and NPY influence each other [20–23], and NPY is regarded as the “stress molecule” [24, 25], which plays an important role in the common core mechanism of psychological and somatic stress responses. However, there is still no systematic study on the mechanism of *ob-R* change in central nervous system under stress and the mechanism of how NPY and *ob-R* regulate appetite and energy metabolism under stress.

The Xiaoyaosan prescription originated from the book *Taiping Huimin Heji Jufang* in the Song Dynasty (960–1127 A.D.) and was composed of eight crude drugs, such as Radix Angelicae Sinensis, Radix Paeoniae Alba, Radix Bupleuri, Rhizoma Atractylodis Macrocephalae, Radix Et Rhizoma Glycyrrhizae, Poria, Rhizoma Zingiberis Recens, and Herba Menthae Haplocalycis. Xiaoyaosan is prescribed to sooth the liver, tonify spleen, and nourish blood. The finished products (pill, decoction, etc.) were always used to treat mental diseases such as depression for centuries in China [26, 27]. Now, they are being used for multiple-system diseases such as mental disorders, neurological diseases, digestive system diseases, respiratory diseases, endocrine diseases, and gynecologic diseases [28–30]. The reliability of the therapeutic effect of Xiaoyaosan in relieving symptoms of chronic stress has been widely proved. For example, Xiaoyaosan can influence the expression of the genes encoding proopiomelanocortin (POMC), corticotropin releasing factor (CRF), encephalin, and preprodynorphin [31, 32]. Xiaoyaosan also reversed chronic immobilization stress- (CIS-) induced decreases in brain-derived neurotrophic factor (BDNF) and increases in tyroxine hydroxylase (TrkB) and neurotrophin 3 (NT-3) in the frontal cortex and the hippocampal CA1 subregion [7]. Xiaoyaosan can interfere with metabolic network abnormalities of chronic unpredictable mild stress or CIS model animals, and we should further seek or elucidate the targets or receptor of characteristic metabolic molecules of antistress effect of drugs [33–35].

Based on the regulating effect of *ob-R* and NPY in the ARC on appetite and energy metabolism and the defined anti-stress effect of Xiaoyaosan, we studied changes in NPY and *ob-R* in the ARC of rats stressed by chronic immobilization in order to elucidate the possible mechanisms of appetite decrease and bodyweight loss under chronic stress. At the same time, we also studied the regulating effect of Xiaoyaosan decoction on the above changes.

## 2. Materials and Methods

### 2.1. Animals

The healthy male Sprague Dawley (SD) rats with bodyweight of 180 ± 20 g were purchased from Beijing Vital River Laboratory Animal Technology Limited Company. Standard animal feeding room: room temperature: 21 ± 1°C; relative humidity: 30% to 40%; Light condition: (light for 12 h: 07:00 to 19:00, darkness for 12 h: 19:00 to 07:00); *ad libitum *purified water. The rats were randomly divided into 4 groups, namely, the control group, the 7-day stress group, the 21-day stress group, and the Xiaoyaosan-treated group, which were also stressed. In each group, there were 24 rats in 8 cages and 3 rats in each cage. The rats in the normal control group were fed routinely for 21 days; continuous immobilization stress for 3 h/day for 7 days was conducted for the rats in the 7-day stress; continuous immobilization stress for 3 h/day for 21 days was conducted for the rats in the 21-day stress groups; while continuous immobilization stress for 3 h/day was conducted for the rats in the Xiaoyaosan-treated group for 21 days. Xiaoyaosan was intragastrically administered 30 min before chronic immobilization stress. The rats were fed and provided with water *ad libitum*. All the animals in the study were maintained in accordance with the guidelines of China legislations on the ethical use and care of laboratory animals. All efforts were made to minimize animal suffering and the number of animals needed to produce reliable data.

### 2.2. Preparation of Extracts of Xiaoyaosan

Composition proportions of Xiaoyaosan prescription: *Poria : Radix Paeoniae Alba : Radix Et Rhizoma Glycyrrhizae : Radix Bupleuri : Radix Angelicae Sinensis : Rhizoma Atractylodis Macrocephalae : Herba Menthae Haplocalycis : Rhizoma Zingiberis Recens* equaled to 3 : 3 : 1.5 : 3 : 3 : 3 : 1 : 1. All 8 crude drugs were purchased from Beijing Tongrentang (Bozhou) Decoction Pieces Limited Company and authenticated by Dr. B. Liu, Department of Botany, and Beijing University of Chinese Medicine. All crude drugs were extracted by the Chinese medicine preparation room of China-Japan Friendship Hospital as previously described [7]. Extraction steps were performed as follows: *Poria, Radix Paeoniae Alba, and Radix Rhizoma Glycyrrhizae* were boiled and extracted three times with 10 volumes (2 h), 8 volumes (1 h), and 8 volumes (1 h) of water to obtain the extraction liquid (A). *Radix Bupleuri, Radix Angelicae Sinensis, Rhizoma Atractylodis Macrocephalae, Herba Menthae, and Rhizoma Zingiberis Recens* were soaked with 10 volumes of water for 12 h to obtain the volatile oil, drug liquid (B), and drug residue (C). Subsequently, C was boiled in 8 volumes of water for 1 h and extracted twice to obtain the extraction solution (D). Extraction solutions A, B, and D were mixed to form the water extraction liquid (E). Next, E was filtered and centrifuged (3000 r/min for 40 min). The supernatant was collected and vacuum dried at 70°C. Then, the dried product and the volatile oil were processed into dry decoction for use. The extraction rate was 18.8%. Xiaoyaosan (was dissolved in deionized water and administered by intragastric injection at a dose of) was 3.854 g/kg*·*d, and deionized water was used in all groups. 

### 2.3. Chronic Immobilization Stress (CIS) Procedure

A previously described chronic constraint method [31] was used in which rats were bound to a binding rack (type T binding platform: the base: width of 10 cm, length of 20 cm, thickness of 2.8 cm; the upper part of binding platform for rat binding: length of 22 cm, maximum width of 6.6 cm; the front end had small frames for fixing the head and small grooves suitable for placing limbs; the upper binding platform had two adjustable soft belts which could, respectively, fix the chest and abdomen of animal) for 3 h every day. Binding time points were randomly selected from 7:00 am to 16:00 pm in an effort to avoid the animal adaptation to a fixed binding time. Moreover, before administration, bodyweight and food intake of rats (including those at 0 day) were weighed. Daily food intake was calculated by subtracting the intraday surplus food amount from the feeding amount at the last one day. 

### 2.4. ELISA for Measurement of Leptin, NPY, and *ob-R* Content in Hypothalamus

On the 22nd day of the trial, 6 rats in the normal control group, the 21-day stress group, and the Xiaoyaosan-treated group were anaesthetized with an intraperitoneal injection 10% chloral hydrate (0.35 to 0.40 mL/100 g bodyweight). Subsequently, the hypothalamus was removed and placed a 2 mL EP tube, immediately frozen on liquid nitrogen (http://www.iciba.com/liquid nitrogen) and stored below −20°C. To create a hypothalamus homogenate: the hypothalamus specimen was boiled in 1 mL normal saline for 3 min. Then, 0.5 mL of 1 N glacial acetic acid was added, and the mixture was homogenized with hand-held electric homogenizer. Next, 0.5 mL of 1 N NaOH was added for neutralization. The solution was mixed uniformly and centrifuged at 3500 ×g for 20 min at 4°C. The supernatant was collected and stored at −20°C. According to the kit instructions, leptin, *ob-R*, and NPY contents in hypothalamus were detected by ELISA method.

### 2.5. Double-Labeling Immunofluorescence for NPY and *ob-R* in the ARC of Hypothalamus

On the 8th day of the trial for the 7-day stress group and on the 22nd day of the trial for the normal control group, the 21-day stress group and Xiaoyaosan-treated group, samples were acquired (the same as following test). 6 rats in each group were anaesthetized with an intraperitoneal injection of 10% chloral hydrate (0.35 to 0.40 mL/100 g bodyweight), and the left ventricular ascending aortic perfusion fixation of the heart was carried out. Firstly, the samples were quickly washed with 0.9% NaCl solution (precooled to 4°C in advance) by perfusion, and then 4% paraformaldehyde solution was perfused with continuously. Perfusion was stopped when the tail tip hardened. Rats were sacrificed by decapitation and the whole brain was taken out. The whole brain was placed into 4% paraformaldehyde solution, stored at 4°C, and fixed for 12 h. Brain tissues were transferred into sucrose solutions with concentration of 20% and 30% for dehydration and stored at 4°C. The constant temperature freezing microtome (Leica CM1900) was used for sectioning, and section thickness was about 30 *μ*m.

The main steps of double-labeling immunofluorescence were as follows: (1) washed with 0.05 M TBS three times, 5 min once; (2) incubated in 0.05 M TBS containing 0.5% TritonX-100 for 1 h in the incubator at 37°C; (3) washed with 0.05 M TBS three times, 5 min each; (4) added 0.05 M TBS blocking liquid containing 10% donkey serum (Millipore Corporation, USA) and 0.5% TritonX-100 and incubated for 1 h at room temperature; (5) removed blocking liquid and adding 1 : 500 of rabbit antineuropeptide Y polyclonal antibody (Millipore Corporation, USA) diluted with 0.05 M TBS containing 2% donkey serum and 0.5%TritonX-100 and incubated at 4°C overnight; (6) washed with 0.05 M TBS containing 2% donkey serum and 0.5% TritonX-100 for three times, 5 min each; (7) added 1 : 200 of Alexa Fluor@ 594 donkey anti-rabbit IgG (Invitrogen, USA, dilution liquid, the same as antibody I) and incubated for 4 h at room temperature, protected from light; (8) washed with 0.05 M TBS for three times, 5 min each; (9) added the blocking liquid (the same as step (4)) and incubated for 1 h at room temperature; (10) removed the blocking liquid and adding 1 : 50 of Goat anti-*ob-R* polyclonal antibody (Santa Cruz Biotechnology, Inc., USA) diluted with 0.05 M TBS containing 2% donkey serum and 0.5% TritonX-100. The mixture was incubated for 40 h at 4°C; (11) washed for three times, 5 min each (the same as step (6)); (12) added 1 : 200 of Alexa Fluor@ 488 donkey anti-goat IgG (Invitrogen, USA, dilution liquid, the same as antibody I). The mixture was incubated for 4 h at room temperature, protected from light; (13) washed with 0.05 M TBS for three times, 5 min each; (14) mounted the section on glass slide, and HardSet Mounting Medium with DAPI (Vector H-1500, USA) was used for sealing. Tris was purchased from Sigma Company; TritonX-100, paraformaldehyde, NaCl, and sucrose were purchased from Beijing Chemical Reagent Limited Company.

ZEISS LSM510 META laser scanning confocal microscope was used for imaging and analysis of 10 slices in each group. The integral optical density (IOD), NPY and *ob-R* colocalization area, and NPY or *ob-R* weight colocalization coefficient were calculated and selected for statistics from analytic results. weight colocalization coefficient represents sum of intensities of colocalizing pixels in channel 1 or 2, respectively, as compared to the overall sum of pixel intensities above threshold and in this channel. Value range of 0-1 (0: no colocalization, 1: all pixels colocalization). The number of positive neurons was measured with Image Pro Plus.

### 2.6. *In Situ* Hybridization for NPY mRNA and *ob-R* mRNA in the ARC

Six rats in each group were injected intraperitoneally with 10% chloral hydrate (0.35 to 0.40 mL/100 g bodyweight), left ventricular ascending aortic perfusion prefixation was carried out, and postfixation of 4% paraformaldehyde was conducted (3 to 8 h). Subsequently, brain tissues were transferred into sucrose solutions with concentration of 20% and 30% for dehydration. The specific steps were the same as in the double-labeling immunofluorescence experiment described above. 0.9% NaCl solution, post-fixation solution of 4% paraformaldehyde, and 20% and 30% sucrose solutions were prepared with RNase-Free water. Constant temperature freezing microtome (Leica CM1900) was used for sectioning, and section thickness was about 14 to 15 *μ*m.

*In situ* hybridization assay kits of rat NPY and *ob-R* were purchased from Wuhan Boster Biological Engineering Limited Company. (1) mRNA sequences of rat *ob-R* target gene: (a) 5′-ATTTT CCACC CAAAA TTCTG ACTAG TGTTG-3′; (b) 5′-ATCTG GCTAT ACAAT GTGGA TCAGG ATCAA-3′; (c) 5′-AAGTT CCTAT GAGAG GGCCT GAATT TTGGA-3′. (2) mRNA sequences of rat NPY target gene: (a) 5′-TACCC CTCCA AGCCG GACAA TCCGG GCGAG-3′; (b) 5′-CTGCG ACACT ACATC AATCT CATCA CCAGA-3′. According to the kit instructions, *in situ* hybridization staining was conducted. All the used solutions were prepared with RNase-Free water. Elite ABC Kit was purchased from Vector, USA; DEPC, Tris, and DAB were purchased from Sigma, USA.

ZEISS Primo Star microscope was used for imaging, and Image-Pro Plus 6.0 Image Analysis System was used for image analysis. Nine slices in each group were selected from the ARC positions (area of 150 *μ*m × 150 *μ*m). In addition, the number of positive cells (cells) and integral optical density (IOD) were counted. IOD = measured value of IOD/total measured area.

### 2.7. RT-qPCR for NPY mRNA and *ob-R* mRNA in the ARC

Six rats in each group were anaesthetized with intraperitoneal injected 10% chloral hydrate (0.35 to 0.40 mL/100 g bodyweight). Brain tissues were rapidly taken out, immediately frozen on liquid nitrogen, and stored at −80°C. Solutions were prepared with DEPC-treated water, and vessels were soaked in DEPC water and sterilized at high temperature.

Oven temperature of the constant temperature freezing microtome (Leica, Germany) was adjusted to −10°C. The section thickness was set as 60 *μ*m (the maximum setting), and shook the hand shank of frozen section to turn back 5 rings with forward 1 ring, then, 300 *μ*m brain tissue was sectioned, and effective tissue slices were selected and placed onto clean glass slides. The ARC was taken out on dry ice with a flat needle, placed into RNase-Free 1.5 mL EP tube, immediately frozen in liquid nitrogen, and stored at −80°C.

Brain tissues were completely homogenized with a hand-held electric homogenizer (KONTES, USA), and the total RNA of the ARC was extracted by using the Trizol method and stored at −70°C. According to the instructions of GoTaq@ 2-Step RT-qPCR System kit (Promega Corporation, USA), reverse transcription reaction and quantitative determination of cDNA were conducted.

According to Genebank sequences and the literatures [36, 37], NPY, *ob-R*, *β*-actin primers were designed. Among them, And *β*-actin acted as the internal reference. Beijing AuGCT DNA-SYN Biotechnology Co., Ltd. was entrusted to synthesize the primers. Primers: NPY, Forward: 5′-TGTGGACTGACCCTCGCTCTAT-3′101, Reverse: 5′-TGTAGTGTCGCAGAGCG GAGTA-3′239, NM_012614.1, 139 bp; *ob-R*, Forward: 5′-TCTGCCTGAAGTTATAGATGATTTG-3′492, Reverse: 5′-GTCACTCCAGACTCCTGAGCCATCC-3′957, NM_012596.1, 466 bp; *β*-actin, Forward: 5′-GCTTCTCTTTAATGTCACGCACG-3′24, Reverse: 5′-CCATCCAGGCTG TGTTGTCC-3′266, NM_031144.2, 243 bp. 

According to GoTaq@ 2-Step RT-qPCR System and “Primed Synthesis Report Sheet” of AuGCT, 25 *μ*L GoTaq@ qPCR Master Mix reaction system was prepared and mixed uniformly. Real-time fluorescent quantitative PCR instrument (Bio-rad Chromo4 CFB-3240, USA) was used for fluorescent quantitative PCR amplification. Optimum conditions of qPCR: NPY & *β*-actin: 95°C 2 min; 95°C 15 s, 60°C 1 min, 40 cycles; 60 to 95°C. *ob-R* & *β*-actin: 95°C 5 min; 95°C 30 s, 59°C 40 s, 72°C 1 min, 30 cycles; 72°C 10 min.

DEPC (diethyl pyrocarbonate), agarose, and Trizol reagent were purchased from Sigma Company, USA; chloroform, isopropyl alcohol, and anhydrous alcohol were purchased from Beijing Chemical Reagent Limited Company.

Fluorescent quantitative PCR results were used to calculate relative expressions of NPY mRNA and *ob-R* mRNA using the previously described of 2^−ΔΔCT^ method [38, 39]. ^ΔΔ^CT = (mean target gene CT value − mean internal reference gene CT value) − (mean reference gene CT value − mean internal gene CT value). Also, *β*-actin acted as the internal reference gene. To calculate the relative expression of each sample (CT), the beta-actin CT value was subtracted from each sample gene CT value. 2^−ΔΔCT^ was used for variance analysis and histogram.

### 2.8. Statistical Processing

Data were expressed as mean ± standard error of mean ($\overset{-}{x}\pm \text{SEM}$). Using SPSS 17.0 software and one-way ANOVA was applied for general data. In addition, LSD method was adopted for the comparisons between groups. Repeated measurement process of general linear model (GLM) in SPSS17.0 was used to conduct one-way ANOVA analysis for repeated measured data (bodyweight and food intake), and multivariate analysis process of variance was used to make comparisons between groups on each time point (LSD method). *P* < 0.05 was considered statistically significant.

## 3. Results

### 3.1. Bodyweight and Food Intake of Rats with Constraint Stress for 21 Days Significantly Reduced; While Xiaoyaosan Prevented This Effect

Before constraint stress, there was no significant difference in bodyweight and food intake among the 3 groups of rats (Table 1). From the 2nd day of constraint stress, bodyweight of simple stress rats was significantly lower than that of the normal control group at the same time point (*P* < 0.05 or *P* < 0.01). From the 6th day of constraint stress, bodyweight of the Xiaoyaosan-treated group was significantly lower than that of the normal control group at the same time point (*P* < 0.05 or *P* < 0.01). From the 16th day to 21st day, bodyweight of the Xiaoyaosan-treated group was significantly higher than that of simple stress rats at the same time point (*P* < 0.05 or *P* < 0.01). 

Table 2 shows that from the 2nd day of constraint stress, food intake of stress rats was significantly lower than that of the normal control group at the same time point (*P* < 0.05 or *P* < 0.01). From the 2nd day to 13th day of constraint stress, food intake of the Xiaoyaosan-treated group was significantly lower than that of the normal control group at the same time point (*P* < 0.05 or *P* < 0.01); while in the 3rd week of constraint stress, the food intake showed an increasing trend. Compared with the control group, there was no significant difference. Particularly on the 17th day, 19th day, and 21th day, the food intake of the Xiaoyaosan-treated group was significantly higher than that of stress rats (*P* < 0.05).

**Figure 1:**
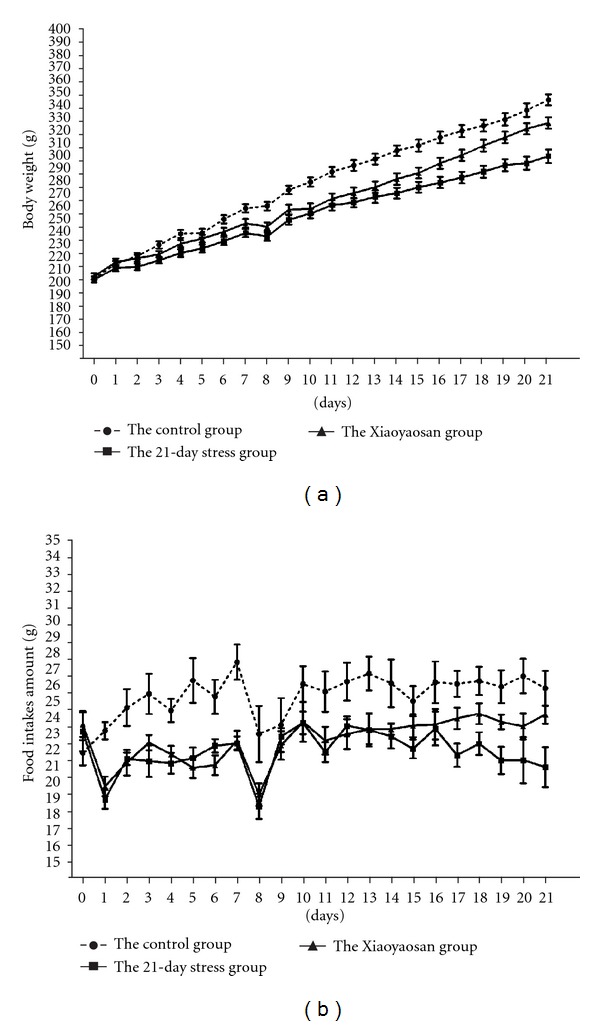
Changes in bodyweight and food intake in three groups. Each point represents the mean values and vertical bars represents SEM.

Figure 1 shows that in the 3rd week of constraint stress, bodyweight and food intake of simple stress rats were significantly lower than those of the normal group and the Xiaoyaosan-treated group. In the 1st week and 2nd week of constraint stress, the bodyweight and food intake of the Xiaoyaosan-treated group was lower than that of the normal control group. In the 3rd week, the bodyweight and food intake significantly increased and showed a trend close to those of the rats of the normal control group.

### 3.2. Hypothalamic Leptin and *ob-R* Protein Expression in the 21-Day Constraint Stress Group Is Significantly Increased; While NPY Expression Shows No Obvious Change

**Figure 2:**
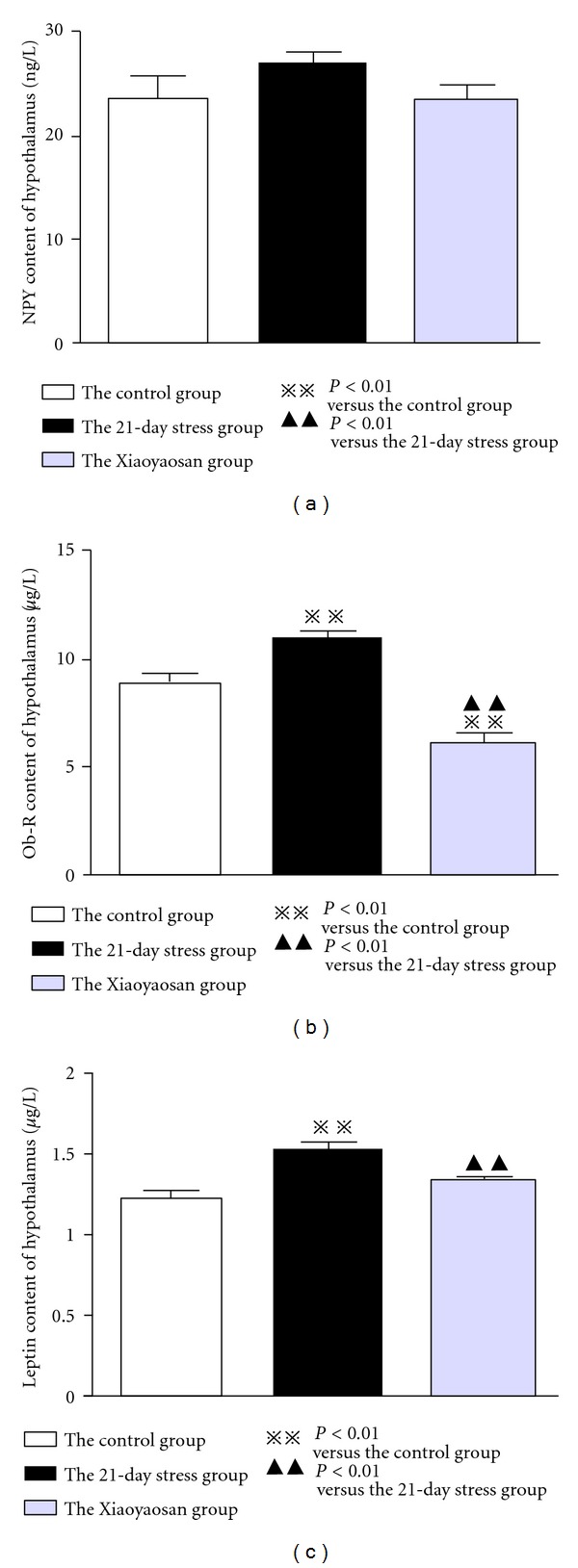
Differences in Leptin, NPY, and *ob-R* contents in hypothalamus. Each bar represents the mean values and vertical bars represent SEM. ^⋇⋇^ *P* < 0.01 as compared with the control group; ^▲▲^ *P* < 0.01 as compared with the 21-day stress group.

For NPY content in rat hypothalamus, among the normal control group (23.715 ± 1.964 ng/L), the 21-day stress group (27.071 ± 1.053 ng/L) and the Xiaoyaosan-treated group (23.463 ± 1.517 ng/L), there were no significant differences (*P* > 0.05) (see Figure 2(a)). *ob-R* expression in rat hypothalamus of the 21-day stress group (10.644 ± 0.311 *μ*g/L) was significantly higher than that of the normal control group (8.798 ± 0.230 *μ*g/L) (*P* < 0.01); while *ob-R* content in rat hypothalamus of the Xiaoyaosan-treated group (5.938 ± 0.421 *μ*g/L) was significantly lower than that of the 21-day stress group and the normal control group, with obvious statistical significance (*P* < 0.01) (see Figure 2(b)). The Leptin content in rat hypothalamus of the 21-day stress group (1.506 ± 0.049 *μ*g/L) was significantly more than that of the normal control group (1.231 ± 0.031 *μ*g/L) (*P* < 0.01); while the Xiaoyaosan-treated group (1.322 ± 0.022 *μ*g/L) was significantly lower than that of the 21-day stress group (*P* < 0.01) (see Figure 2(c)).

### 3.3. Double-Labeling Immunofluorescence Results Show That *ob-R* Protein Expression in the ARC of the 21-Day Group Is More Than That of the Normal Group; While NPY Protein Expression Has No Obvious Change

**Figure 3:**
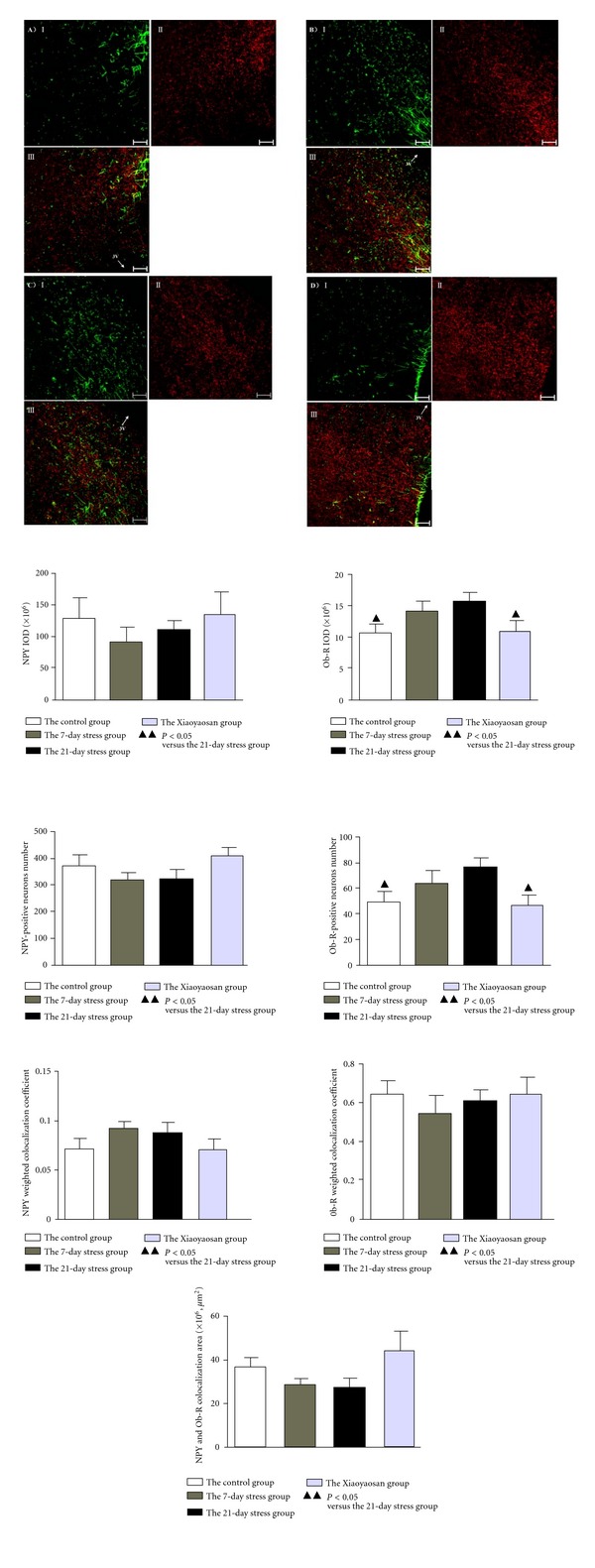
Double immunofluorescence for NPY and *ob-R* in the ARC. (A–D) Representative images of NPY, *ob-R*, and colocalization in ARC. (A) The control group. (B) The 7-day stress group. (C) The 21-day stress group. (D) The Xiaoyaosan-treated group. In each figure, *ob-R* was labeled with green fluorescence (I), NPY was labeled with red fluorescence (II), and co-loalization of NPY and *ob-R* was double-labeled in yellow (III). Scale bars = 50 *μ*m for all images. NPY and *Ob-R* colocalization area, NPY or *Ob-R* IOD, and weighted colocalization coefficient were analyzed with Zeiss LSM Image Examiner, and the number of positive neurons was measured with Image Pro Plus. Each bar represents the mean values and vertical bars represent SEM. ^▲^ *P* < 0.05 as compared with the 21-day stress group. 3V means the third ventricle.

NPY neurons were labeled with red fluorescence (II), widely distributed in the form of granules or block mass; while *ob-R* neurons were labeled as green fluorescence (I). Yellow staining sites were double-staining neurons of *ob-R* and NPY (III). Microscopic observation shows that compared to the control group, *ob-R*-positive cells in the ARC of the 7-day stress group and the 21-day stress group are increased; while positive expression neurons of NPY are reduced. Compared to the stress groups, *ob-R*-positive expression cells in the ARC of the Xiaoyaosan-treated group are reduced; while positive expression neurons of NPY are increased (see Figure 3). 

Semiquantitative statistical analysis shows that for NPY IOD and positive neurons number in the ARC, the 7-day stress group (IOD: 91.452 ± 23.361, ×106; positive neurons number: 319.500 ± 31.201) and the 21-day stress group (IOD: 109.685 ± 15.836, ×106; positive neurons number: 323.700 ± 37.392) are different from the normal control group (IOD: 128.389 ± 33.972, ×106; positive neurons number: 372.000 ± 42.848) and the Xiaoyaosan-treated group (IOD: 134.692 ± 36.194, ×106; positive neurons number: 411.400 ± 31.219), but there is no significant difference (*P* > 0.05) among the 4 groups. The *ob-R* IOD and positive neurons number in the ARC of the 21-day stress group (IOD: 15.710 ± 1.683, ×106; positive neurons number: 76.100 ± 6.602) is significantly higher than that of the normal control group (IOD: 10.734 ± 1.444, ×106; positive neurons number: 49.400 ± 8.275) and the Xiaoyaosan-treated group (IOD: 11.114 ± 1.734, ×106; positive neurons number: 46.200 ± 8.779), *P* < 0.05. 

Among 4 groups of double-labeling immunofluorescence staining, there is no significant difference (*P* > 0.05) in colocalization area (the normal control group: 36.995 ± 5.089, ×102, *μ*m^2^; the 7-day stress group: 28.073 ± 5.100, ×102, *μ*m^2^; the 21-day stress group: 27.291 ± 3.098, ×102, *μ*m^2^; the Xiaoyaosan-treated group: 44.237 ± 9.341, ×102, *μ*m^2^), NPY weighted colocalization coefficient (the normal control group: 0.071 ± 0.011; the 7-day stress group: 0.091 ± 0.009; the 21-day stress group: 0.088 ± 0.009; the Xiaoyaosan-treated group: 0.071 ± 0.009) or *ob-R* weighted colocalization coefficient (the normal control group: 0.654 ± 0.066; the 7-day stress group: 0.548 ± 0.099; the 21-day stress group: 0.616 ± 0.056; the Xiaoyaosan-treated group: 0.656 ± 0.082) (see Figure 3).

### 3.4. *In Situ* Hybridization Results Show That Compared with the Control Group, Positive Expression of *ob-R* mRNA in the ARC of Stress Rats Is Significantly Increased; While Positive Expression of NPY mRNA Is Unchanged with Stress Time

**Figure 4:**
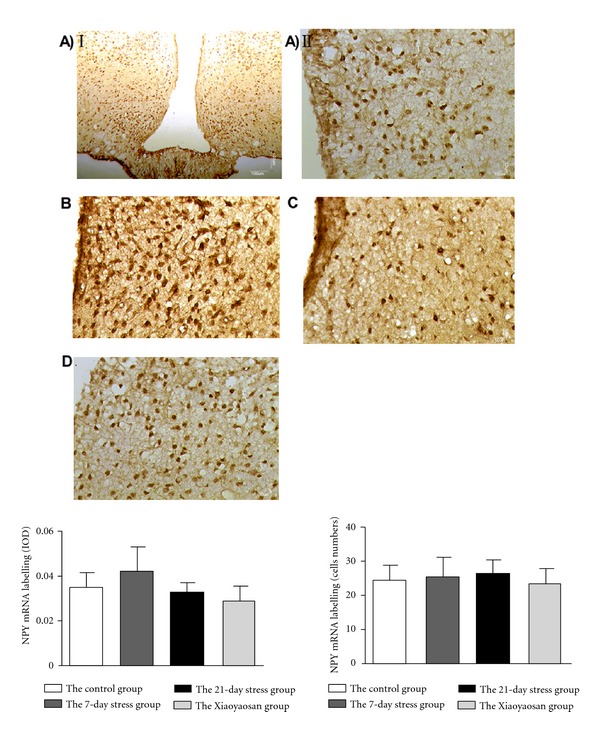
*In situ* hybridization for NPY mRNAin the ARC. (A–D) Representative images of NPY mRNA in ARC. (A) The control group. (B) The 7-day stress group. (C) The 21-day stress group. (D) The Xiaoyaosan-treated group. Scale bars = 100 *μ*m for A (I) and 10 *μ*m for other images. IOD of NPY mRNA labeling and cells of NPY mRNA labeling were measured with Image Pro Plus. Each bar represent the mean values and vertical bars represent SEM.

Combination of *in situ* hybridization staining image analysis and semiquantitative statistical analysis shows that the positive expression of NPY mRNA of the 7-day stress group is obviously more than that of the normal control group. However, among the 4 groups, there is no significant difference between positive expression IOD and cells of NPY mRNA (see Figure 4). NPY mRNA IOD: the normal control group 0.035 ± 0.006, the 7-day stress group 0.042 ± 0.011, the 21-day stress group 0.045 ± 0.010, and the Xiaoyaosan-treated group 0.029 ± 0.006. NPY mRNA cell numbers: the normal control group 24.333 ± 5.427, the 7-day stress group: 25.500 ± 5.513, the 21-day stress group 26.278 ± 3.896, and the Xiaoyaosan-treated group 23.722 ± 3.761.

**Figure 5:**
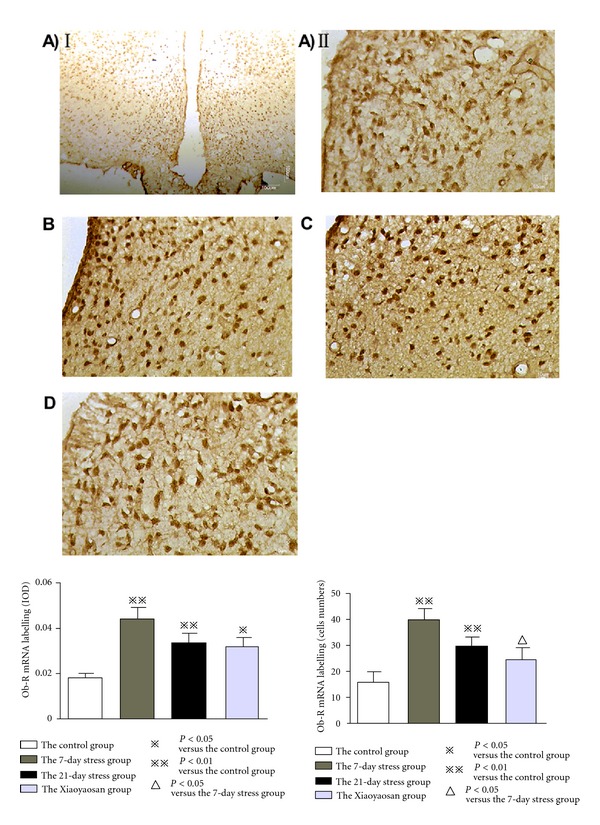
*In situ* hybridization for *ob-R* mRNAin the ARC. (A–D) Representative images of *ob-R* mRNA in ARC. (A) The control group. (B) The 7-day stress group. (C) The 21-day stress group. (D) The Xiaoyaosan-treated group. Scale bars = 100 *μ*m for A (I) and 10 *μ*m for other images. IOD of *ob-R* mRNA labeling and cells of *ob-R* mRNA labeling were measured with Image Pro Plus. Each bar represent the mean values and vertical bars represent SEM. ^⋇^ *P* < 0.05 or ^⋇⋇^ *P* < 0.01 as compared with the control group; ^Δ^ *P* < 0.05 as compared with the 7-day stress group.

*ob-R* mRNA localized to the cytoplasm and nucleus of the normal control group and the Xiaoyaosan-treated group, while the 7-day stress group and the 21-day stress group mainly have nucleus expressions, especially for rats with constraint stress for 21 days. Semiquantitative analysis suggests that the positive expression IOD of *ob-R* mRNA of the 7-day stress group (0.044 ± 0.005), 21-day stress group, (0.034 ± 0.005), and the Xiaoyaosan-treated group (0.032 ± 0.005) is significantly more than that of the normal control group (0.019 ± 0.002) (*P* < 0.05 or *P* < 0.01). The positive *ob-R* mRNAs in the 7-day stress group (40.333 ± 5.014) and the 21-day stress group (30.889 ± 4.191) were significantly increased, compared with those of the normal control group (16.111 ± 4.470), *P* < 0.05 or *P* < 0.01; while those of the Xiaoyaosan-treated group (25.500 ± 3.623) were significantly reduced, compared than those of the 7-day stress group (*P* < 0.05) (see Figure 5).

### 3.5. RT-qPCR Results Show That Compared with the Normal Control Group, Relative Expression of NPY mRNA in the ARC of Stress Rats Is Significantly Reduced; While Relative Expression of *ob-R* mRNA Is Significantly Increased

**Figure 6:**
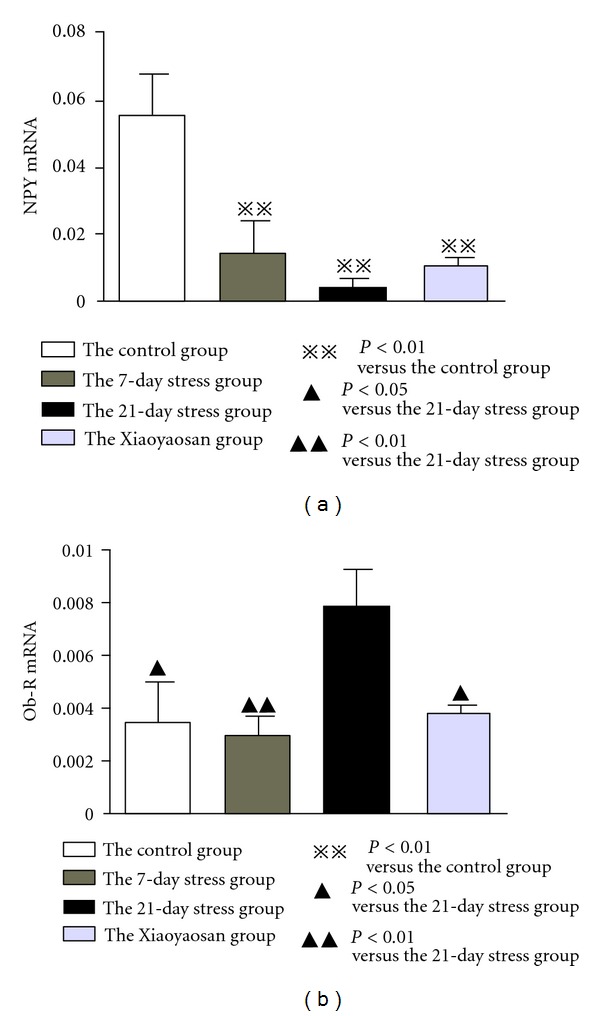
RT-qPCR detection of NPY mRNA and *ob-R* mRNA in the ARC. To calculate the relative amount of NPY or *ob-R* gene expression the formula (ΔCT = CT_target  gene_ − CT_internal  reference_) is used and analyzed with 2^−ΔCT^ for statistics. Each bar represent the mean values and vertical bars represent SEM. ^⋇⋇^ *P* < 0.01 as compared with the control group; ^▲^ *P* < 0.05 or ^▲▲^ *P* < 0.01 as compared with the 21-day stress group.

Relative expression of NPY mRNA and *ob-R* mRNA was calculated using the 2^−ΔΔCT^ method. As for relative expression of NPY mRNA, compared with the normal control group, the relative expression of the 7-day stress group was 0.113 (coefficient of variation (CV): 0.021 to 0.593), the 21-day stress group was 0.038 (CV: 0.009 to 0.156), and the Xiaoyaosan-treated group was 0.213 (CV: 0.098 to 0.463). Compared with the 21-day stress group, the relative content of NPY mRNA of the Xiaoyaosan-treated group was 5.540 (CV: 1.535 to 20.000). This suggests that compared with the normal control group, relative expression in NPY mRNA of the 7-day stress group, the 21-day stress group, and the Xiaoyaosan-treated group was significantly reduced as shown in Figure 6 (*P* < 0.01).

Compared with the normal control group, relative content of *ob-R* mRNA in the 7-day stress group was 0.950 (CV: 0.287 to 3.150), that in the 21-day stress group was 2.969 (CV: 1.161 to 7.595), and that of the Xiaoyaosan-treated group was 1.286 (CV: 0.480 to 3.447). Compared with the 21-day stress group, relative content of *ob-R* mRNA of the normal control group was 0.337 (CV: 0.132 to 0.862), that of the 7-day stress group was 0.320 (CV: 0.130 to 0.786), and that of the Xiaoyaosan-treated group was 0.433 (CV: 0.241 to 0.778). Figure 6 shows that relative expression of *ob-R* mRNA of the 21-day stress group was significantly increased compared with that of the normal control group (*P* < 0.05); while relative expression of the Xiaoyaosan-treated group was significantly reduced compared with that of the 21-day stress group (*P* < 0.05).

## 4. Discussion

In this study, bodyweight and food intake of stressed rats increased more slowly than the normal rats with lengthening of chronic immobilization stress, in compliance with previous literatures and the previous research results of this research team [2–8]; while Xiaoyaosan can ameliorate the above changes.

The hypothalamus contains massive neuropeptide nervous pathways that promote appetite (NPY/Agouti-related peptide (AgRP)) and neuropeptide nervous pathways that inhibite appetite (POMC/Cocaine-amphetamine regulated transcript (CART)). These nerves that promote appetite and inhibit appetite are sent by the ARC and projected onto other nucleus groups/brain areas of the hypothalamus, such as the lateral hypothalamic area (LHA), ventromedial nucleus (VMN), dorsomedial nucleus (DMN), and paraventricular nucleus (PVN), constituting the “appetite regulation network” (ARN) [40–42]. The ARN is the key the central system regulating food intake and bodyweight balance. Peripherally secreted appetite regulation signals, such as leptin, can cross the blood-brain barrier to reach hypothalamus nucleus group and influence appetite by affecting these two types of peptides [43].

Leptin is a polypeptide hormone secreted mainly by white adipose tissues. A number of electrophysiological and behavioral studies show that leptin regulates food intake and energy homeostasis mainly by means of the central nervous system (especially hypothalamus) [41, 44]. Also, hypothalamus is the main target of leptin [45].

*ob-R* is a high-affinity receptor of leptin, belonging to the class I cytokine super family. Current studies suggest that *ob-R* gene has at least 6 (a to f) splicing isomers [46]. Among them, *ob-R*b is a long receptor, and it is the main functional receptor. *ob-R*b is mainly expressed in the hypothalamus, and it has limited expression in peripheral tissues [47, 48]. Therefore, *ob-R*b is the main action receptor of leptin.

Leptin binds with *ob-R* in the hypothalamus, which can play a role in inhibiting appetite, reducing energy intake and increasing energy expenditure. Therefore, this study firstly detected leptin and *ob-R* contents in hypothalamus. The results show that compared with the control group, leptin and *ob-R* contents in the hypothalamus of the 21-day stress group are significantly increased, which suggests that CIS can increase Leptin levels in the hypothalamus and stimulate an increase in *ob-R* (mainly *ob-R*b) expression, and the combination specificities of the two damages the balance of hypothalamus “appetite regulation network.”

As *ob-R* (mainly *ob-R*b) is widely expressed on the ARC, VMN, DMN, PVN, periventricular nucleus. and neurons in the lateral hypothalamic area [10, 11]; these areas are the main areas for regulating food intake and bodyweight. Among them, the ARC in the bottom of the third ventricle plays an important role in energy metabolism regulation by leptin [49]. For NPY/AgRP neuron and POMC/CART neuron in the ARC, the former can promote appetite, inhibit energy expenditure, and inhibit degradation of alpha-melanocyte-stimulating hormone (*α*-MSH); the later-secreted POMC can inhibit appetite and promote energy expenditure. The binding of leptin and *ob-R* can inhibit generation of NPY/AgRP neurons and release of NPY and AgRP. On the other hand, it can promote generation of POMC neuron and release of POMC and thus regulates the body's energy metabolism [50]. This study further detected *ob-R* protein and gene expressions in the ARC of stress rats. The results show that constraint stress (especially stress for 21 days) can induce *ob-R* protein and gene expressions in the ARC to be significantly higher than those of normal rats, which suggests that greatly increased leptin in the hypothalamus binding to *ob-R* in the ARC disrupts the homeostasis of NPY/AgRP-expressing neurons and POMC/CART-expressing neurons that regulate food intake and energy metabolism.

NPY is one of the most expressed neuropeptides in central nervous tissues, distributed in brain tissues and spinal cord but not the cerebellum. As a neurotransmitter, neurohormone, and neuromodulator, NPY is involved in the complexity of stress response. It not only regulates the emotional and behavioral changes caused by stress, such as anxiety and depression, but also promotes appetite mainly by means of the hypothalamus. Also, NPY neurons are abundant in the hypothalamus, especially in the ARC [9, 51].

Studies show that after 8 weeks of moderate psychological stress, NPY expression in the hypothalamus paraventricular nucleus, arcuate nucleus, and other areas of rats is significantly reduced [19], and NPY expression in the hippocampus area and ARC of rats receiving CIS for 21 days is reduced [52]. This study detected NPY content in the hypothalamus of rats receiving constraint stress by ELISA and observed NPY protein and gene expression in the ARC of restrained rats by means of immunofluorescence and *in situ* hybridization. Although there were changes in NPY protein and gene expressions, compared with the normal rats, the differences were not statistically significant. While RT-qPCR results show that NPY mRNA expression in the ARC of CIS rats is significantly reduced, in line with previous results [19, 52]. We hypothesize that after chronic stress, the binding of leptin with *ob-R* in the ARC possibly causes a decrease in NPY expression and inhibits appetite to reduce food intake and thus reduces the increase in bodyweight.

However, many studies show that while food intake of chronically stressed rats is reduced, NPY mRNA expression in the arcuate nucleus is increased. For example, Sergeyev et al. [53] investigated the rats exposed to repeated, unpredictable, and mild stress for 3 weeks. As a result, NPY mRNA expression in the arcuate nucleus significantly increased; while the expression on hippocampal dentate gyrus was reduced [53]. Makino et al. proved that for acute stress (2 h) or chronic repeated immobilization stress (2 h daily, for 14 days), NPY mRNA expression in the arcuate nucleus was significantly increased [54]. The inconsistent results of NYP mRNA expression in the ARC induced by chronic stress: (1) As a “stress molecule” [24, 25], NPY and the HPA axis interact [20–23]. Psychological stress can activate the HPA axis to promote an increase in glucocorticoid secretion.* In vitro* and *in vivo* experimental studies show that glucocorticoid can stimulate the neurons in ARC to synthesize NPY, and there are response fragments upstream of the NPY gene encoding region carrying glucocorticoid [55]. This could possibly explain the significant increase of NPY mRNA expression in the hypothalamus after psychological stress. (2) Additional reports show that increases in blood glucose can upregulate NPY expression in hypothalamus [56]. Therefore, an increase in the of body's blood glucose after stress is possibly another explanation for the significant increase of NPY mRNA in hypothalamus. (3) The significant increase of NPY mRNA in hypothalamus after psychological stress is possibly associated with a neuropeptide and neurotransmitter secretion disorder caused by psychological stress, but it is necessary to carry out further research.

The results of this study from double-labeling immunofluorescence, *in situ* hybridization, and RT-qPCR demonstrate that *ob-R* protein and gene expression in the ARC of CIS rats are significantly increased. While various observation methods show that NPY protein and gene expressions have inconsistent changes (there were differences only for RT-qPCR assay), suggesting that the intermediate link of rat bodyweight is decreased and food intake loss of rats caused by the binding of leptin with *ob-R* in hypothalamus does not mainly pass through the NPY nervous inhibition pathway, but it possibly passes through another nerve pathway (possibly POMC, etc.).

Immobilization is a widely used method of nerve stimulation in stressed animal models. Immobilization stress deprives an animal of freedom of activity and is similar to the human psychosomatic disease process. It is an example of psychological frustration stress [57], mainly embodied in body's emotional disorder and behaviors such as depression, anxiety, and abnormal changes in appetite and bodyweight. Traditional Chinese prescription Xiaoyaosan is used to treat emotional disorders such as depression, anxiety, and irritation, and some symptoms such as dizziness, head fullness, dry eye, sense of pharyngeal foreign body, chest and hypochondrium distending pain, two-hypochondrium distending pain, chest and hypochondrium dull pain, breast distending pain, epigastric fullness discomfort after eating, spiritlessness, languidness, sigh, premenstrual irritability, menstrual abdominal distending pain, sexual dysfunction, bad sleep, stool dry pond, lusterless complexion (http://dict.cnki.net/dict_result.aspx?searchword=%E9%9D%A2%E8%89%B2%E5%B0%91%E5%8D%8E&tjType=sentence&style=&t=lusterless+complexion), premenstrual breast distending pain, premenstrual chest and hypochondrium distending pain, menstrual breast distending pain, and small and wiry pulse and has functions of dispersing stagnated liver Qi for relieving Qi stagnation and nourishing blood and strengthening spleen, suitable for liver depression and spleen deficiency syndrome [30, 58]. It has been proven through systematical evaluation that for the treatment of depression, Xiaoyaosan is effective and has no side effects [28]. Therefore, Xiaoyaosan has become a prescription preferred by domestic scholars for resisting chronic stress, and its chronic stress resistance has been widely researched. Also, a certain progress has been achieved [7, 31–35]. Therefore, we studied the effectiveness of selected extraction of Xiaoyaosan as an intervention to regulate NPY and *ob-R* in the ARC of stress rats. We show through immunofluorescence staining, *in situ* hybridization, or RT-qPCR, that Xiaoyaosan decreases *ob-R* protein and gene expression in the ARC of stressed rats.

## 5. Conclusion

The results of this study show that for somatic discomfort symptoms such as appetite decrease and bodyweight loss under chronic stress, increase of *ob-R* expression in the ARC is possibly a central neuroendocrine mechanism. Also, leptin signaling through *ob-R* in hypothalamus does not appear to utilize the NPY nervous inhibition pathway, and we presume that it possibly passes through another nervous pathway (possibly POMC, etc.). Therefore, the molecular basis of decreased regulation of *ob-R* expression in the ARC and intracellular signal transduction pathway requires further research. In addition, the results of this study suggest that a decrease in *ob-R* in the ARC is possibly the target of Xiaoyaosan, regulating somatic discomfort states with appetite decrease and weight loss induced by chronic stress.

## Figures and Tables

**Table 1:** Changes of bodyweight ($g,\;\;\bar{x}\;\pm \;\;$SEM).

Day	The control group	The 21-day stress group	The Xiaoyaosan group
Day 0	211.167 ± 1.989	210.750 ± 2.122	213.080 ± 2.256
Day 1	222.375 ± 2.302	219.208 ± 2.430	223.560 ± 2.619
Day 2	228.083 ± 2.417	220.125 ± 2.366*	226.520 ± 2.684
Day 3	236.458 ± 2.685	224.958 ± 2.401**	229.480 ± 2.331*
Day 4	244.625 ± 2.985	230.417 ± 2.969**	237.400 ± 2.682
Day 5	245.167 ± 3.170	233.833 ± 3.002**	241.080 ± 2.666
Day 6	255.417 ± 3.033	239.250 ± 2.928**	246.280 ± 2.846*
Day 7	263.667 ± 3.154	245.208 ± 3.092**	252.440 ± 3.181*
Day 8	265.250 ± 3.168	242.625 ± 3.139**	250.040 ± 3.098**
Day 9	277.250 ± 3.227	255.083 ± 3.528**	262.640 ± 3.550**
Day 10	283.125 ± 3.483	259.708 ± 3.470**	263.160 ± 4.052**
Day 11	290.708 ± 3.685	265.917 ± 3.782**	270.720 ± 3.954**
Day 12	295.375 ± 3.978	267.917 ± 3.618**	275.040 ± 4.121**
Day 13	300.042 ± 3.999	272.167 ± 4.720**	279.280 ± 4.336**
Day 14	306.333 ± 4.059	274.792 ± 4.113**	285.360 ± 4.331**
Day 15	310.167 ± 4.370	279.167 ± 4.039**	289.800 ± 4.115**
Day 16	316.208 ± 4.258	282.708 ± 4.206**	297.040 ± 4.112^∗∗▲^
Day 17	320.958 ± 4.519	286.458 ± 4.276**	302.880 ± 4.151^∗∗▲^
Day 18	324.875 ± 4.473	290.792 ± 4.391**	310.120 ± 4.278^∗▲▲^
Day 19	329.542 ± 4.592	295.625 ± 4.455**	316.200 ± 4.279^∗▲▲^
Day 20	336.375 ± 4.996	297.125 ± 4.804**	322.680 ± 4.165^∗▲▲^
Day 21	344.083 ± 4.132	302.208 ± 4.942**	326.920 ± 4.209^∗▲▲^

*N*_the control group_ = 24, *N*_the 21-day stress group_ = 24, *N*_the xiaoyaosan group_ = 24.

*P < 0.05, **P < 0.01 versus the control group; ^▲^ *P* < 0.05, ^▲▲^ *P* < 0.01 versus the 21-day stress group.

**Table 2:** Changes of food intake (*N* = 8, *g*,   $\bar{x}\;\pm \;\;$SEM).

Day	The control group	The 21-day stress group	The Xiaoyaosan group
Day 0	22.556 ± 0.733	23.794 ± 1.160	24.188 ± 0.715
Day 1	23.825 ± 0.498	19.856 ± 0.551**	20.594 ± 0.575**
Day 2	25.162 ± 1.067	22.200 ± 0.359*	21.981 ± 0.736**
Day 3	25.963 ± 1.165	22.056 ± 0.908**	23.138 ± 0.445*
Day 4	25.000 ± 0.678	21.940 ± 0.592**	22.456 ± 0.488**
Day 5	26.744 ± 1.293	22.248 ± 0.602**	21.681 ± 0.608**
Day 6	25.794 ± 0.978	22.950 ± 0.391**	21.831 ± 0.563**
Day 7	27.806 ± 1.011	23.088 ± 0.409**	23.275 ± 0.548**
Day 8	23.644 ± 1.631	19.440 ± 0.725*	20.119 ± 0.672*
Day 9	24.163 ± 1.570	23.502 ± 0.476	22.975 ± 0.832
Day 10	26.531 ± 1.032	24.279 ± 0.660	24.337 ± 1.153
Day 11	26.094 ± 1.173	22.575 ± 0.567*	23.250 ± 0.802*
Day 12	26.669 ± 1.093	24.115 ± 0.540*	23.631 ± 0.860*
Day 13	27.131 ± 0.977	23.877 ± 0.968*	23.919 ± 0.871*
Day 14	26.575 ± 1.394	23.502 ± 0.707*	23.931 ± 0.342
Day 15	25.544 ± 0.877	22.771 ± 0.544*	24.113 ± 0.656
Day 16	26.631 ± 1.198	23.954 ± 0.986	24.194 ± 0.876
Day 17	26.538 ± 0.751	22.403 ± 0.673**	24.544 ± 0.622^▲^
Day 18	26.713 ± 0.811	23.081 ± 0.660**	24.812 ± 0.606
Day 19	26.381 ± 0.943	22.103 ± 0.793**	24.337 ± 0.425^▲^
Day 20	26.988 ± 1.000	22.125 ± 1.338**	24.079 ± 0.757
Day 21	26.281 ± 1.008	21.719 ± 1.164**	24.763 ± 0.528^▲^

*P < 0.05, **P < 0.01 versus the control group; ^▲^ *P* < 0.05 versus the 21-day stress group.
